# COVID-19 vaccine preferences among university students in Hong Kong: a discrete choice experiment

**DOI:** 10.1186/s13104-021-05841-z

**Published:** 2021-11-22

**Authors:** Xue Li, Man Yui Chong, Ching Yui Chan, Vindy Wing Sum Chan, Xinning Tong

**Affiliations:** 1 Department of Medicine, Li Ka Shing Faculty of Medicine, The University of Hong Kong, Hong Kong, People’s Republic of China; 2 Centre for Safe Medication Practice and Research, Department of Pharmacology and Pharmacy, Li Ka Shing Faculty of Medicine, The University of Hong Kong, Hong Kong, People’s Republic of China; 3 Li Ka Shing Faculty of Medicine, University of Hong Kong, Hong Kong, People’s Republic of China

**Keywords:** COVID-19 vaccine, Preference and vaccination decision, Discrete choice experiment

## Abstract

**Objective:**

To promote public health and resume university activities, COVID-19 vaccination has been mandated from an increasing number of universities worldwide. The objective of the study is to understand the factors that impact preference and willingness to take the vaccine among university students in Hong Kong universities utilizing an online questionnaire. The findings will be imperative for health education and the success of the vaccination program.

**Results:**

We conducted a discrete choice experiment survey among university students in Hong Kong and applied conditional logit regression to estimate their vaccine preference and the weight of each attribute. Regression results showed adverse reactions, efficacy, origin of the vaccine, required number of doses and out-of-pocket price are significant determinants for the choice of vaccine, ranked from the most to least important. Similar preference weighting results were observed after adjusting age, sex, monthly household income, studying medical-related subjects and recent influenza vaccination. Safety, efficacy and origin of the vaccine are key drivers for vaccination decisions among young adults in Hong Kong. Health education and communication focused on these factors are urgently needed to overcome vaccine hesitancy and improve the vaccine uptake.

**Supplementary Information:**

The online version contains supplementary material available at 10.1186/s13104-021-05841-z.

## Introduction

As of 25 June 2021, over 2.7 billion doses of COVID-19 vaccines have been administered globally as urgent use [1]. To promote public health and resume campus activities, increasing numbers of higher education worldwide have announced mandatory COVID-19 vaccination before the new semester begins. However, the younger adult population, alongside other socioeconomic factors, has been consistently reported to be less inclined to receive the vaccine [2]. We conducted a cross-sectional survey to understand COVID-19 vaccine preferences among university students in Hong Kong, with the aim of informing health education to improve vaccine uptake.

## Main text

### Methods

We designed orthogonal-based discrete choice experiment (DCE), which incorporated six COVID-19 vaccine attributes (Additional file 1: Table S1) into 18 choice scenarios (Additional file 1: Table S2), to reveal COVID-19 vaccine preferences among university students. The survey questionnaire was developed for this study (Additional file 2). Data was collected using web-based survey tool Qualtrics (Qualtrics, Provo, UT) between 25th January and 25th February 2021—the date before the Hong Kong Government officially launched the mass vaccination scheme. The online questionnaire was distributed by snowballing methods through social media and university mass emails. Conditional logit regression was used to estimate the preference weight of each attribute [3]. Ethics approval was obtained from Institutional Review Board of the University of Hong Kong/Hospital Authority Hong Kong West Cluster (UW-20-792).

### Results

**Fig. 1:**
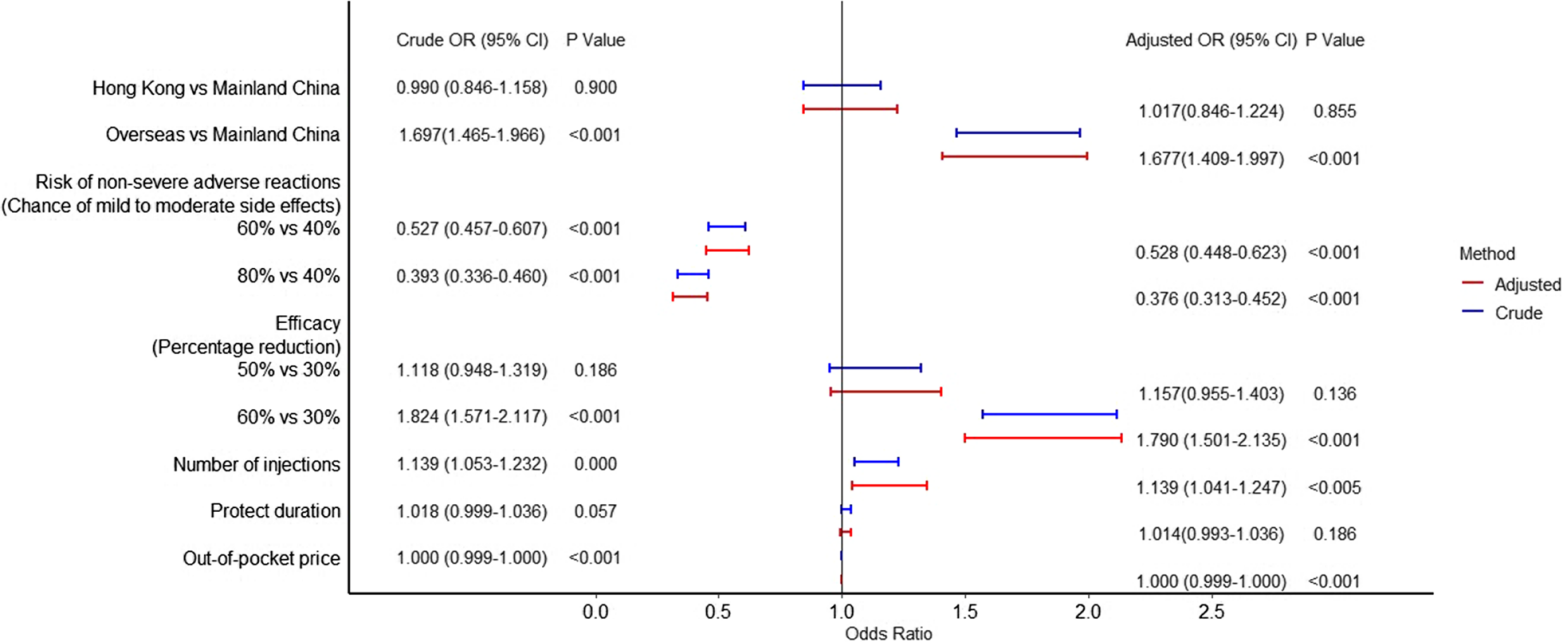
COVID-19 vaccine preference attributes among university students in Hong Kong. odds ratio > 1 suggests higher likelihood of selecting the vaccine over the reference profile # adjusted by age, sex, monthly household income, recent influenza vaccination, studying medical-related subjects

We received 194 (71.1%) completed DCE responses from 270 respondents in eight University Grants Committee-funded institutions in Hong Kong. The participants were predominately aged 18–22 years, of which 60% are studying medical-related subjects (Additional file 1: Table S3). As indicated by the regression coefficient, non-severe adverse reactions, efficacy, origin of the vaccine, required number of doses and out-of-pocket price are significant determinants for the choice of vaccine, ranked from the most to least important. Adjusted by age, sex, monthly household income, studying medical-related subjects and recent influenza vaccination, the model yielded similar preference weighting results. Safety, efficacy, and origin of the vaccine remained the top three influential factors for vaccine preferences in both models (Fig. 1). Studying medical-related subjects [Odds Ratio (OR) 0.987; 95% Confidence Interval (CI) 0.858–1.136] or having received influenza vaccine recently [1.026 (0.887–1.188)] showed marginal impact in the decision process (data not shown).

### Discussion

In line with the global vaccination recommendation to university students, almost all Hong Kong higher educations have recently announced COVID-19 vaccination as a condition for campus residency, which requires all students and staff to take the vaccine or have regular testing [4]. This is a crucial time to understand vaccine preferences among university students to optimize health education and vaccine supply. In the current study, vaccine safety, efficacy and origin are the most influential determinants of vaccine choices among the university students in Hong Kong, independent of demo-socioeconomic status, medical knowledge, and influenza vaccination. Students are willing to have vaccines with less adverse reactions, higher efficacy, and more doses to achieve a good immune response. Of note, vaccines originating from overseas, but not Hong Kong or Mainland China, are highly preferred with over 65% increased likelihood of taking the vaccine. The results are consistent with previous studies showing that perceived risk-benefits of the vaccine, confidence in the healthcare system and vaccine manufacturers are key drivers for vaccine acceptance [5, 6]. This further highlights an imperative need to convey evidence-based safety and efficacy information for all available vaccines in the licensed region. Persistent effort should be made to foster confidence in government and manufacturers to improve vaccine supply and acceptance.

### Conclusion

University students in Hong Kong preferred vaccines with superior safety and efficacy profiles and vaccines manufactured overseas. Transparent evidence showing the safety and efficacy/effectiveness of vaccines from different origins is urgently needed to overcome vaccine hesitancy among university students in Hong Kong.

## Limitations

The discrete choice survey was designed in September 2021, before the availability of data from COVID-19 vaccine clinical trials. Therefore, the tested ranges of safety and efficacy may not match the benchmark trial data. Also, this is a cross-sectional study conducted before the launch of massive COVID-19 vaccination program in Hong Kong. University student’s perception and preference of COVID-19 vaccine might have changed under the circumstance of more real-world evidence emerging afterwards. In addition, the willingness-to-pay item may not be applicable to countries/regions where the vaccine has been provided for free. Nevertheless, cost is the least consideration in our discrete choice survey. Main factors of COVID-19 vaccine preference among Hong Kong university students are safety, efficacy profiles and origins of the vaccine, hence we anticipate this will not affect the conclusion significantly. Longitudinal studies with multiple follow-ups and evidence-based questionnaire design are highly encouraged to investigate the preference changes and the consequential policy implications.

## Supplementary Information

**Additional file 1**: **Table S1.** Covid-19 vaccine attributes and levels used in the discrete choice experiment design. **Table S2.** Discrete choice experiment survey (example). **Table S3.** Characteristics of participants (N, %).

**Additional file 2.** Full version of online questionnaire.

## Data Availability

The datasets used and analyzed during the current study are available from the corresponding author on reasonable request.

## Abbreviations

DCE: Discrete choice experiment
OR: Odds ratio
CI: Confidence interval
