# Supplementary material for: COVID-19 vaccine preferences among university students in Hong Kong: a discrete choice experiment
Source: BMC Res Notes. 2021 Nov 22;14:421. doi: 10.1186/s13104-021-05841-z (PMC8607213; doi:10.1186/s13104-021-05841-z)
Supplement: Supplementary file 1 — Additional file 1: Table S1. Covid-19 vaccine attributes and levels used in the discrete choice experiment design. Table S2. Discrete choice experiment survey (example). Table S3. Characteristics of participants (N, %). [file 13104_2021_5841_MOESM1_ESM.docx]

COVID-19 vaccine preferences among university students in Hong Kong: a discrete choice experiment

Additional file 1

Three tables

Table S1 Covid-19 vaccine attributes and levels used in the discrete choice experiment design

Table S2 Discrete choice experiment survey (example)

Table S3 Characteristics of participants (N, %)

**Table S1 Covid-19 vaccine attributes and levels used in the discrete choice experiment design**

| **Attributes** | **Description** | **Levels** |
| --- | --- | --- |
| Efficacy | Percentage reduction of a disease in a vaccinated group vs unvaccinated | 30%  50%  70% |
| Protection duration | Duration of protection provided by the full initial regimen without later booster doses | 10 Years  5 Years  3 Years  2 Years  1 Year  6 months |
| Risk of non-severe adverse effects | Chance of mild to moderate side effects occurring (e.g. headache, injection site pain, muscle pain, fatigue, impaired appetite) | 40%  60%  80% |
| Area of origin | Area of origin | Local (Hong Kong)  Mainland  Overseas |
| Out-of-pocket price | Total price (in HK$) (out of pocket payment) for all the doses needed for the initial regimen | HK$800  HK$400  HK$0 |
| Number of injections | Number of injections needed for the original regimen to establish abovementioned efficacy | 1  2  3 |

**Table S2 Discrete choice experiment survey (example)**

Below is a discrete choice experiment including 18 scenarios per set for COVID-19 vaccines. Each participant will need to provide answers to 6 scenarios. Each scenario will include two different unlabeled COVID-19 vaccines with different levels of attributes. Participants have to select the option in that scenario that is more preferable for them. (Below is one sample scenario).

| **Scenario 3** | **Option A** | **Option B** |
| --- | --- | --- |
| Efficacy | 30% | 50% |
| Protection duration | 6 months | 1 year |
| Risk of non-severe adverse effects | 40% | 60% |
| Area of origin | Overseas | Mainland |
| Out-of-pocket price (HK$ to complete the full vaccination schedule) | 0 | 400 |
| Number of Injections | 1 | 2 |
| Please select [X] the vaccine you prefer |  |  |

**Table S3. Characteristics of participants (N, %)**

|  | Total  (N = 194) | Studying medical-related subjects (N=117) | Studying non-medical-related subjects (N=77) | |
| --- | --- | --- | --- | --- |
| Age (years) | | | |  |
| 18-22 | 166 (85.5) | 93 (79.5) | 73 (94.8) | |
| 23-27 | 25 (12.9) | 21 (17.9) | 4 (5.2) | |
| ≥28 | 3 (1.5) | 3 (2.5) | 0 (0) | |
| Male | 82 (42.3) | 31 (26.5) | 39 (50.6) | |
| Asian ethnicity | 193 (99.5) | 116 (99.1) | 77 (100) | |
| Monthly household income (HK$ per month) | | | |  |
| <20000 | 19 (9.8) | 13 (11.1) | 6 (7.8) | |
| 20000-39999 | 33 (17.0) | 14 (12.0) | 19 (25) | |
| 40000-59999 | 32 (16.5) | 21 (17.9) | 11 (14) | |
| 60000-79999 | 21 (10.8) | 12 (10.2) | 9 (12) | |
| 80000-99999 | 9 (4.6) | 9 (7.7) | 0 (0) | |
| >100000 | 27 (13.9) | 19 (16.2) | 8 (10) | |
| Not reported | 53 (27.3) | 29 (24.8) | 24 (31.2) | |
| Recent influenza vaccination | 119 (61.3) | 85 (72.6) | 34 (44.2) | |
