# Supplementary material for: COVID-19 vaccine preferences among university students in Hong Kong: a discrete choice experiment
Source: BMC Res Notes. 2021 Nov 22;14:421. doi: 10.1186/s13104-021-05841-z (PMC8607213; doi:10.1186/s13104-021-05841-z)
Supplement: Supplementary file 2 — Additional file 2. Full version of online questionnaire. [file 13104_2021_5841_MOESM2_ESM.docx]

**Acceptance and Perception of COVID-19 Vaccines Among University Students in Hong Kong: a Discrete Choice Experiment**

香港大學生對於新型冠狀病毒疫苗的接受程度和看法：離散選擇實驗

**Online Questionnaire, 19 Oct 2020, Version 1.0**

網上問卷調查，2020年10月19日，版本1.0

**Name of Researchers 研究員名單 :** Chong Man Yui, Chan Ching Yui, Chan Wing Sum Vindy, Chan Yan Yee, Chau Ying Hei, Chung Tin Kit Jonathan, Tsang Yuen Ying, Tse Tze Yung, Wong Tsz Ying

**Background 背景**

Regarding the COVID-19 pandemic, it has already lasted for almost a year. Recently, at least 80 vaccines are under investigation and development. However, none of them are ready for use due to safety issues. In view of this, we wish to study the effect of medical knowledge in the perception and decision-making of vaccination and preferences among university students. The following questionnaire will be used to obtain the above mentioned information.

新型冠狀病毒的疫情已持續近一年。近來數月，各界都在努力研發新的疫苗，到目前為止已有至少80款疫苗是處於發展階段。但是由於安全問題，暫時沒有任何一款疫苗是可供大眾使用的。所以，我們想藉著這個研究，探討香港大學生擁有基本醫學知識與否對其在選擇疫苗、接受疫苗的程度和看法有沒有影響。而下面的問卷將會用來獲取上述資料。

**Withdrawal from study 退出調查**

Participants can freely withdraw from the study by contacting our principal investigator Mr. Chong Man Yui by email at u3558104@hku.hk. All data collected will be anonymous and all data collected from participants who have withdrawn from the study will be discarded and eradicated immediately.

所有參加者都有權利隨時中斷或退出研究。如參加者有意中斷或退出研究，可直接

聯繫我們的首席研究員莊文睿先生（電郵：u3558104@hku.hk）。我們會以匿名的方式收集所有資料，如有參加者在中途中斷或退出研究，我們會即時將其資料銷毀。

**Confidentiality 保密性**

All responses received will be kept strictly confidential and only the research investigators and authorised third-party can access the data. The information will be kept for up to 5 years and will be destroyed afterwards. For any query, please consult the Privacy Commissioner for Personal Data (Tel No. 2827 2827) for the regulated monitoring and supervision of personal data protection as to assure a comprehensive awareness and understanding of the significance of complying with the law governing privacy data.

我們會對收集回來的所有資料保密，只有研究員和被授權的第三方才有權利去讀取資料。所有資料只會保存5年，5年後，所有資料將會完全被銷毀。為了全面保障對遵守私隱條例的重要性的意識，如有任何關於監察和管理個人資料的事宜，請向個人資料私隱專員查詢 （電話：2827 2827）。

**Subject Informed Consent Form（知情同意書）**

**Title of Project:** Acceptance and Perception of COVID-19 Vaccines Among University Students in Hong Kong: a Discrete Choice Experiment

項目名稱：香港大學生對於新型冠狀病毒的接受程度和看法：離散選擇實驗

# **Name of Researcher （**研究員名單）: Chong Man Yui, Chan Ching Yui, Chan Wing Sum Vindy, Chan Yan Yee, Chau Ying Hei, Chung Tin Kit Jonathan, Tsang Yuen Ying, Tse Tze Yung, Wong Tsz Ying

Please put your initial inside the box

(請在方格內填上姓名的首字母)

1. I confirm that I have read and understood the information sheet 口

dated __/__/__ for the above study and have had the opportunity

to ask questions.

我確認我已閱讀並明白以上研究（日期___/____/____）的資料表，

我亦有提出問題的權利。

2. I understand that my participation is voluntary and that I am free 口

to withdraw at any time, without giving any reason, without my

medical care or legal rights being affected.

我明白這是一個自願性參於的研究，我可以在沒有任何原因及在健康

和法定權利都沒受影響下，隨時中斷或退出研究。

3. I understand where it is relevant to my taking part in research, I 口

give permission for these individuals to have access to my

responses.

我明白在研究中我所參與的部分，並允許上述提及的研究員和

被授權的第三方去讀取我的資料。

4. I agree to take part in the above study.

我同意參與以上的研究。

口

__________________ ___________ _______________

## *Name of subject* （參與者名字）

__________________

*Researcher*  (研究員）

*Date* （日期） *Signatu*re （簽署）

*___________* _______________

*Date*（日期） *Signature* （簽署）

**This questionnaire includes *FOUR* sections. Estimated time to complete is 12 minutes.**

該問卷包括四個部分。估計完成時間為12分鐘。

**Section A. Demographics 人口統計資料**

1. What is your gender? 你的性別是甚麼?

- Male 男性
- Female 女性
- Other其他

1. What is your age group 你的年齡組別是甚麼?

- 18-22 years old 18-22歲
- 23-27 years old 23-27歲
- 28-34 years old 28-34歲
- 35 years old or above 35歲或以上

1. Which ethnicity best describe you? 哪一個種族最適合形容你?

- Asian 亞洲人
- White 白人Black or African American
- 黑人或非洲裔美國人Hispanic or Latino
- 西班牙裔或拉丁裔Native American or American Indian
- 美國原住民或美國印第安人
- Other 其他

1. Which university are you currently studying in? 你正在就讀香港哪所大學？

- The University of Hong Kong 香港大學
- The Chinese University of Hong Kong 香港中文大學
- The Hong Kong University of Science and Technology 香港科技大學
- The Hong Kong Polytechnic University 香港理工大學
- City University of Hong Kong 香港城市大學
- Hong Kong Baptist University 香港浸會大學
- Lingnan University 嶺南大學
- The Education University of Hong Kong 香港教育大學
- The Open University of Hong Kong 香港公開大學
- The Hang Seng University of Hong Kong 香港恒生大學
- Hong Kong Shue Yan University 香港樹仁大學
- Others 其他

1. Which programme are you currently in? 你正在就讀大學哪類課程?

- Undergraduate programmes 本科課程
- Taught postgraduate programmes 修課式研究生課程
- Research postgraduate programmes 研究式研究生課程

1. Which subject are you studying now? 你正在學習那一學科?

- Medicine 醫科
- Dental Surgery 牙科
- Chinese Medicine 中醫
- Nursing 護理學
- Mental Health Nursing 精神健康護理學
- Pharmacy 藥劑
- Biomedical Sciences 生物醫學
- Public Health 公共衛生
- Medical Laboratory Science 醫療化驗科學
- Biomedical Engineering 生物醫學工程
- Radiography 放射學
- Occupational Therapy 職業治療學
- Physiotherapy 物理治療學
- Optometry 眼科視光學

## Veterinary Medicine 獸醫學

- Human biology 人類生物學
- Others medical-related courses 其他醫療相關課程
- Others (non-medical-related courses) 其他 (非醫療相關課程)

1. Apart from your current subject, what other university subjects did you study before? 除了目前的科目外, 你以前有否學習大學裏其他學科?

- I did not study any other university subject before 我以前沒有學習大學的其他學科
- Medicine 醫科
- Dental Surgery 牙科
- Chinese Medicine 中醫
- Nursing 護理學
- Mental Health Nursing 精神健康護理學
- Pharmacy 藥劑
- Biomedical Sciences 生物醫學
- Public Health 公共衛生
- Medical Laboratory Science 醫療化驗科學
- Biomedical Engineering 生物醫學工程
- Radiography 放射學
- Occupational Therapy 職業治療學
- Physiotherapy 物理治療學
- Optometry 眼科視光學

## Veterinary Medicine 獸醫學

- Human biology 人類生物學
- Others medical-related courses 其他醫療相關課程
- Others (non-medical-related courses) 其他 (非醫療相關課程)

1. What is your father’s highest education level? 您父親的最高學歷是甚麼?

- Primary school or below 小學或以下
- Secondary school 中學
- Associate Degree / Diploma 副學士 / 文憑
- Degree or above 學位或以上
- Refuse to answer 拒絕回答

1. What is your mother’s highest education level? 您母親的最高學歷是甚麼?

- Primary school or below 小學或以下
- Secondary school 中學
- Associate Degree / Diploma 副學士 / 文憑
- Bachelor's Degree or above 學士學位或以上
- Refuse to answer 拒絕回答

1. Which of the following best describe your father’s occupation? 以下哪項最適合描述您父親的職業？

- Accounting 會計
- Banking/ Finance 銀行/金融
- Property management 物業管理
- Management/Administration 管理/行政
- Marketing Representative/Sales 營業代表/售貨員
- Customer Service客戶服務
- Catering/Hotel/Tourism 餐飲/酒店/旅遊
- Clerk文員
- Legal services 法律服務
- Medical services醫療服務
- Education 教育
- Lab/Research & development 實驗/研究與開發
- Engineering /Construction/Survey 工程/建築/測量
- Design/ Draftsworker設計/繪圖
- Media/advertising/Entertainment媒體/廣告/娛樂
- Computer and information technology 電腦及資訊科技
- Community and social service 社區及社會服務
- Transportation/logistics 運輸/物流
- Production/Factory 生產/工廠職業
- Installation/Maintenance 安裝/維修
- Agriculture/forestry/fishing 農業/林業/漁業
- Technician 技工
- Retired 已退休
- Others 其他
- Refuse to answer 拒絕回答

1. Which of the following best describe your mother’s occupation? 以下哪項最適合描述您母親的職業？

- Accounting 會計
- Banking/ Finance 銀行/金融
- Property management 物業管理
- Management/Administration 管理/行政
- Marketing Representative/Sales 營業代表/售貨員
- Customer Service客戶服務
- Catering/Hotel/Tourism 餐飲/酒店/旅遊
- Clerk文員
- Legal services 法律服務
- Medical services醫療服務
- Education 教育
- Lab/Research & development 實驗/研究與開發
- Engineering /Construction/Survey 工程/建築/測量
- Design/ Draftsworker設計/繪圖
- Media/advertising/Entertainment媒體/廣告/娛樂
- Computer and information technology 電腦及資訊科技
- Community and social service 社區及社會服務
- Transportation/logistics 運輸/物流
- Production/Factory 生產/工廠職業
- Installation/Maintenance 安裝/維修
- Agriculture/forestry/fishing 農業/林業/漁業
- Technician 技工
- Housewife 家庭主婦
- Retired 已退休
- Others 其他
- Refuse to answer 拒絕回答

1. What is your monthly household income (in HKD)? 你的家庭月收入是多少 (港幣)？

- < $20000
- $20000 - $39999
- $40000 - $59999
- $60000 - $79999
- $80000 - 99999
- > $100000
- Do not wish to tell 拒絕回答

1. How many family members do you live with (excluding yourself)? 你與多少個家庭成員住在一起（不包括你自己）？

- 1
- 2
- 3
- 4
- 5
- >5

1. Have you been vaccinated with the influenza vaccine before? 你曾否接種過流感疫苗?

- Yes 有
- No 否

**Section B. Attitude towards vaccines 對於疫苗的看法**

**Please read each statement below and select the description** *(Strongly agree/ Agree/ Neither agree or disagree/ Disagree/ Strongly disagree)* **that matches your opinion the most.**

**請閱讀下文以選擇適當的答案 (非常同意/同意/中立/不同意/非常不同意)**

1. Media allegations about the connection between vaccines with chronic diseases, such as autism and multiple sclerosis, have led me to doubt about vaccination as a safe method.

根據不同傳媒報道，注射疫苗可以引致不同的長期疾病，例如自閉症和多發性硬化，令我懷疑注射疫苗是否一種安全的防疫措施

2. It is important to keep vaccination coverage of the population in order to avoid the emergence of new epidemics.

高參與率的疫苗注射計劃對於防範任何新的疫情爆發十分重要

3. Educating parents about vaccines is an important way to connect a vaccination coverage of the population
教育家長關於疫苗的知識對於能否有高參與率的疫苗注射計劃是非常重要

4. There is not enough evidence that immunization prevents the occurrence of infectious diseases
現時沒有足夠的證據或文獻顯示疫苗注射可以防止傳染病傳播

5. Pharmaceutical companies promote vaccination of children for profit, although they are aware of the fact that it is harmful.
不同的製藥公司即使知道疫苗注射有機會傷害到小孩的身體，但仍然鼓勵小孩接種疫苗來獲取利益

6. It is a social obligation to get vaccinated for an epidemic disease
在疫情爆發中接種疫苗是一種公民責任

7. It is safer to be vaccinated for a disease than stay idle
接種任何疫苗相比起完全不接種疫苗更安全

8. You would vaccinate your children with all the childhood vaccines offered under the governmental scheme
你會選擇讓你的小孩接種全部政府兒童免疫接種計劃下的疫苗

9. You would get the COVID-19 vaccine when it is available

當新型冠狀病毒疫苗研發成功，你會選擇去接種這個疫苗

10. You would advise your family members to get vaccinated for COVID-19

你會建議你的家人去接種新型冠狀病毒的疫苗

**Section C. Knowledge on vaccines 對於疫苗的知識**

**You are provided with 10 statements below. You are required to label each statement as “True” or “False”, or “Do not know” if you do not know the answer or are unsure.
以下有10種不同的說法，你需要判斷每一個說法是 “屬實” 或 “虛假” 。若果你對於某一個說法不肯定或不明白可以選擇 “不知道” 為答案**

1. Vaccines are not necessary as we have treatments for infections (eg. Antibiotics, antivirals) (FALSE)
   因為現時有好多治療傳染病的方法（例如抗生素）所以注射疫苗並不必要
2. Smallpox was eradicated successfully due broadly applied vaccination programs (TRUE) 天花可以成功被消除是因為相關的疫苗己被廣泛接種
3. The efficacy of vaccines has been scientifically proven (TRUE)
   已經有相關科學文獻證明疫苗的效能
4. People would be more resistant towards infectious diseases if they were not vaccinated against all diseases (FALSE)
   假如一般人不接種全部疫苗，會對傳染病有更高的免疫能力
5. Vaccination may trigger chronic diseases like autism, multiple sclerosis and diabetes (FALSE)
   接種疫苗有機會引致不同的長期疾病例如糖尿病，多發性硬化及自閉症
6. The immune system may be overloaded through multiple vaccinations (TRUE)
   人體的免疫系統會因過多的疫苗注射而超負荷
7. Vaccination at early childhood will impair the development of the immune system (FALSE)
   在小孩早期發育階段接種疫苗會影響免疫系統的發展
8. The chemicals included in vaccines are not harmful to humans (FALSE)
   在疫苗中包含的化學成分對人體是無害的
9. Vaccination will increase the occurrence of allergies (FALSE)
   注射疫苗有較大機會引致過敏反應
10. Vaccinations are 100% effective (FALSE)
    疫苗注射是100%有效

**Section D: Preferences for COVID-19 vaccines 對於新型冠狀病毒疫苗的看法**

**Below are descriptions of different attributes of a COVID-19 vaccine that is applied in this section 以下有各方面對於新型冠狀病毒疫苗的資料**

| Attributes 性質 | Description 詳述 |
| --- | --- |
| Efficacy 效能 | Percentage reduction of a disease in a vaccinated group versus unvaccinated group  接種疫苗組別和未接種疫苗組別之間的疾病減少百分比 |
| Duration of protection 疫苗有效時長 | Duration of protection provided by the full initial regimen 接種初期疫苗療程後的總有效時長 |
| Risk of adverse effects 併發症風險 | Chance of mild to moderate side effects occurring (eg. Headache, injection site pain, muscle pain, fatigue, impaired appetite) 有機會引致輕微至中等的副作用  例如頭痛,肌肉酸痛,疲倦,食欲不振,注射部位疼痛 |
| Area of origin 原產地 | The area at which the vaccine was manufactured. 生產疫苗的地方 |
| Number of injections 接種疫苗次數 | Number of injections (within a year) needed for the initial regimen to establish abovementioned efficacy 達到初期疫苗療程理想效果的接種次數 （1年內） |
| Total price 價錢 | Total price (in HKD) (out of pocket payment) for all the injections needed  注射疫苗所需金額 (港幣) |

**Section D1. Attribute Ranking 性質排列**

**Rank the above attributes of vaccines based on their importance to you. (From most important to least important). ). 是根據重要性排列以上各項性質**

**（1是最重要，6是最不重要）**

1.

2.

3.

4.

5.

6.

**Section D2. Discrete Choice experiment 情景題**

Below is a discrete choice experiment including 18 scenarios per set for COVID-19 vaccines. Each participant will need to provide answers to 6 scenarios. Each scenario will include two different COVID-19 vaccines with different levels of attributes. Participants have to select the option in that scenario that is more preferable for them. (Below are three sample scenarios)
以下的問題包含18個關於新冠狀肺炎疫苗的情景，每個情景提供兩個新冠狀肺炎疫苗的資料，試於各情景中選出較佳的選項。

**Block A:**

| Scenario 1 out of 6 情境1 | Option A | Option B |
| --- | --- | --- |
| Efficacy 效能 | 50% | 30% |
| Duration 疫苗有效時長 | 10 years 10年 |  |
| Risk of adverse effects併發症風險 | 40% | 60% |
| Area of origin 原產地 | Overseas 外國 | Local(HK) 香港 |
| Price 價錢 (HKD) | $800 | $0 |
| Number of Injections接種疫苗次數 | 5 years 5年 | 2 |
| Please select [X] the vaccine you prefer |  |  |

| Scenario 2 out of 6 情境 2 | Option A | Option B |
| --- | --- | --- |
| Efficacy 效能 | 70% | 50% |
| Duration 疫苗有效時長 | 6 months 6個月 | 6 months 6個月 |
| Risk of adverse effects併發症風險 | 80% | 40% |
| Area of origin 原產地 | Local(HK) 香港 | Overseas 外國 |
| Price 價錢 (HKD) | $800 | $800 |
| Number of Injections接種疫苗次數 | 3 | 2 |
| Please select [X] the vaccine you prefer |  |  |

| Scenario 3 out of 6 情境3 | Option A | Option B |
| --- | --- | --- |
| Efficacy 效能 | 30% | 50% |
| Duration 疫苗有效時長 | 6 months 6個月 | 1 year 1年 |
| Risk of adverse effects併發症風險 | 40% | 60% |
| Area of origin 原產地 | Overseas 外國 | Mainland 中國大陸 |
| Price 價錢 (HKD) | $0 | $400 |
| Number of Injections接種疫苗次數 | 1 | 2 |
| Please select [X] the vaccine you prefer |  |  |

| Scenario 4 out of 6 情境4 | Option A | Option B |
| --- | --- | --- |
| Efficacy 效能 | 30% | 30% |
| Duration 疫苗有效時長 | 3 years 3年 | 1 year 1年 |
| Risk of adverse effects併發症風險 | 60% | 40% |
| Area of origin 原產地 | Local(HK) 香港 | Overseas 外國 |
| Price 價錢 (HKD) | $0 | $0 |
| Number of Injections接種疫苗次數 | 2 | 1 |
| Please select [X] the vaccine you prefer |  |  |

| Scenario 5 out of 6 情境5 | Option A | Option B |
| --- | --- | --- |
| Efficacy 效能 | 70% | 70% |
| Duration 疫苗有效時長 | 10 years 10年 | 1 year 1年 |
| Risk of adverse effects併發症風險 | 60% | 80% |
| Area of origin 原產地 | Mainland 中國大陸 | Local(HK) 香港 |
| Price 價錢 (HKD) | $0 | $800 |
| Number of Injections接種疫苗次數 | 3 | 3 |
| Please select [X] the vaccine you prefer |  |  |

| Scenario 6 out of 6 情境6 | Option A | Option B |
| --- | --- | --- |
| Efficacy 效能 | 30% | 70% |
| Duration 疫苗有效時長 | 5 years 5年 | 6 months 6個月 |
| Risk of adverse effects併發症風險 | 80% | 60% |
| Area of origin 原產地 | Mainland 中國大陸 | Mainland 中國大陸 |
| Price 價錢 (HKD) | $800 | $0 |
| Number of Injections接種疫苗次數 | 2 | 3 |
| Please select [X] the vaccine you prefer |  |  |

**Block B:**

| Scenario 1 out of 6 情境1 | Option A | Option B |
| --- | --- | --- |
| Efficacy 效能 | 50% | 50% |
| Duration 疫苗有效時長 | 3 years 3年 | 10 years 10年 |
| Risk of adverse effects併發症風險 | 80% | 40% |
| Area of origin 原產地 | Overseas外國 | Local(HK) 香港 |
| Price 價錢 (HKD) | $400 | $0 |
| Number of Injections接種疫苗次數 | 3 | 3 |
| Please select [X] the vaccine you prefer |  |  |

| Scenario 2 out of 6 情境2 | Option A | Option B |
| --- | --- | --- |
| Efficacy 效能 | 50% | 30% |
| Duration 疫苗有效時長 | 2 years 2年 | 3 years 3年 |
| Risk of adverse effects併發症風險 | 80% | 60% |
| Area of origin 原產地 | Mainland 中國大陸 | Overseas外國 |
| Price 價錢 (HKD) | $0 | $800 |
| Number of Injections接種疫苗次數 | 1 | 3 |
| Please select [X] the vaccine you prefer |  |  |

| Scenario 3 out of 6 情境3 | Option A | Option B |
| --- | --- | --- |
| Efficacy 效能 | 50% | 70% |
| Duration 疫苗有效時長 | 6 months 6個月 | 2 years 2年 |
| Risk of adverse effects併發症風險 | 60% | 90% |
| Area of origin 原產地 | Mainland 中國大陸 | Overseas外國 |
| Price 價錢 (HKD) | $400 | $0 |
| Number of Injections接種疫苗次數 | 2 | 2 |
| Please select [X] the vaccine you prefer |  |  |

| Scenario 4 out of 6 情境4 | Option A | Option B |
| --- | --- | --- |
| Efficacy 效能 | 30% | 30% |
| Duration 疫苗有效時長 | 1 year 1年 | 10 years 10年 |
| Risk of adverse effects併發症風險 | 40% | 80% |
| Area of origin 原產地 | Mainland 中國大陸 | Mainland 中國大陸 |
| Price 價錢 (HKD) | $400 | $800 |
| Number of Injections接種疫苗次數 | 3 | 2 |
| Please select [X] the vaccine you prefer |  |  |

| Scenario 5 out of 6 情境5 | Option A | Option B |
| --- | --- | --- |
| Efficacy 效能 | 50% | 70% |
| Duration 疫苗有效時長 | 1 year 1年 | 3 years 3年 |
| Risk of adverse effects併發症風險 | 60% | 40% |
| Area of origin 原產地 | Local(HK) 香港 | Local(HK) 香港 |
| Price 價錢 (HKD) | $800 | $400 |
| Number of Injections接種疫苗次數 | 1 | 2 |
| Please select [X] the vaccine you prefer |  |  |

| Scenario 6 out of 6 情境6 | Option A | Option B |
| --- | --- | --- |
| Efficacy 效能 | 70% | 70% |
| Duration 疫苗有效時長 | 2 years 2年 | 10 years 10年 |
| Risk of adverse effects併發症風險 | 40% | 60% |
| Area of origin 原產地 | Local(HK) 香港 | Overseas外國 |
| Price 價錢 (HKD) | $400 | $400 |
| Number of Injections接種疫苗次數 | 2 | 1 |
| Please select [X] the vaccine you prefer |  |  |

**Block C:**

| Scenario 1 out of 6 情境1 | Option A | Option B |
| --- | --- | --- |
| Efficacy 效能 | 70% | 30% |
| Duration 疫苗有效時長 | 5 years 5年 | 6 months 6個月 |
| Risk of adverse effects併發症風險 | 60% | 80% |
| Area of origin 原產地 | Overseas外國 | Local(HK) 香港 |
| Price 價錢 (HKD) | $400 | $400 |
| Number of Injections接種疫苗次數 | 1 | 1 |
| Please select [X] the vaccine you prefer |  |  |

| Scenario 2 out of 6 情境2 | Option A | Option B |
| --- | --- | --- |
| Efficacy 效能 | 70% | 50% |
| Duration 疫苗有效時長 | 3 years 3年 | 2 years 2年 |
| Risk of adverse effects併發症風險 | 40% | 60% |
| Area of origin 原產地 | Mainland 中國大陸 | Local(HK) 香港 |
| Price 價錢 (HKD) | $800 | $800 |
| Number of Injections接種疫苗次數 | 1 | 1 |
| Please select [X] the vaccine you prefer |  |  |

| Scenario 3 out of 6 情境3 | Option A | Option B |
| --- | --- | --- |
| Efficacy 效能 | 70% | 50% |
| Duration 疫苗有效時長 | 1 year 1年 | 5 years 5年 |
| Risk of adverse effects併發症風險 | 80% | 80% |
| Area of origin 原產地 | Overseas外國 | Overseas外國 |
| Price 價錢 (HKD) | $0 | $400 |
| Number of Injections接種疫苗次數 | 2 | 3 |
| Please select [X] the vaccine you prefer |  |  |

| Scenario 4 out of 6 情境4 | Option A | Option B |
| --- | --- | --- |
| Efficacy 效能 | 30% | 50% |
| Duration 疫苗有效時長 | 10 years 10年 | 3 years 3年 |
| Risk of adverse effects併發症風險 | 6% | 80% |
| Area of origin 原產地 | Local(HK) 香港 | Mainland 中國大陸 |
| Price 價錢 (HKD) | $400 | $0 |
| Number of Injections接種疫苗次數 | 1 | 1 |
| Please select [X] the vaccine you prefer |  |  |

| Scenario 5 out of 6 情境5 | Option A | Option B |
| --- | --- | --- |
| Efficacy 效能 | 50% | 30% |
| Duration 疫苗有效時長 | 5 years 5年 | 2 years 2年 |
| Risk of adverse effects併發症風險 | 40% | 40% |
| Area of origin 原產地 | Local(HK) 香港 | Mainland 中國大陸 |
| Price 價錢 (HKD) | $0 | $400 |
| Number of Injections接種疫苗次數 | 3 | 3 |
| Please select [X] the vaccine you prefer |  |  |

| Scenario 6 out of 6 情境6 | Option A | Option B |
| --- | --- | --- |
| Efficacy 效能 | 30% | 70% |
| Duration 疫苗有效時長 | 2 years 2年 | 5 years 5年 |
| Risk of adverse effects併發症風險 | 60% | 40% |
| Area of origin 原產地 | Overseas外國 | Mainland 中國大陸 |
| Price 價錢 (HKD) | $800 | $800 |
| Number of Injections接種疫苗次數 | 3 | 1 |
| Please select [X] the vaccine you prefer |  |  |

**Appendix 1: Details of the attributes and their corresponding levels**

| Attributes | Description | Levels |
| --- | --- | --- |
| Efficacy | Percentage reduction of a disease in a vaccinated group versus unvaccinated group | 30%  50%  70% |
| Duration of protection | Duration of protection provided by the full initial regimen | 10 Years  5 Years  3 Years  2 Years  1 Year  6 months |
| Risk of adverse effects | Chance of mild to moderate side effects occurring (eg. Headache, injection site pain, muscle pain, fatigue, impaired appetite) | 40%  60%  80% |
| Country of origin | Country of origin | Local (HK)  Mainland  Overseas |
| Number of injections | Number of injections (within a year) needed for the initial regimen to establish abovementioned efficacy | 1  2  3 |
| Total price | Total price (out of pocket payment) for all the injections needed | HK$800  HK$400  HK$0 |

**References:**

Cvjetkovic, S. J., Jeremic, V. L., & Tiosavljevic, D. V. (2017). Knowledge and attitudes toward vaccination: A survey of Serbian students. *Journal of Infection and Public Health,* *10*(5), 649-656. doi:10.1016/j.jiph.2017.05.008

Zingg A, Siegrist M. Measuring people's knowledge about vaccination: developing a one-dimensional scale. Vaccine. (2012); 30(25):3771-7. doi: 10.1016/j.vaccine.2012.03.014. Epub 2012 Mar 20. PMID: 22445808.
